# A Retrospective Study of the Seroreactivity of Screening Markers for Transfusion-Transmissible Infections in Blood Donors at Sunderlal Patwa Government Medical College and District Hospital, Mandsaur, India

**DOI:** 10.7759/cureus.97932

**Published:** 2025-11-27

**Authors:** Santosh Jayant, Vartika Mishra, Harshul Patidar

**Affiliations:** 1 Pathology, Amaltas Institute of Medical Sciences, Dewas, IND; 2 Pathology, Ruxmaniben Deepchand Gardi Medical College, Ujjain, IND; 3 Pathology, Sunderlal Patwa Government Medical College, Mandsaur, IND

**Keywords:** blood bank, blood donors, hepatitis b, hiv, prevalence of reactive markers, retrospective study, sunderlal patwa government medical college, syphilis, transfusion transmissible infections, tti screening seroreactivity

## Abstract

Background: Transfusion-transmissible infections (TTIs) pose a major risk to the safety of blood transfusions, with diseases such as HIV, hepatitis B virus (HBV), hepatitis C virus (HCV), and syphilis being of particular concern. Routine screening of blood donors is crucial for minimizing the risk of transmission. This retrospective study was conducted to assess the seroprevalence of reactive screening markers for TTIs among blood donors at the Indira Gandhi District Hospital associated with Sunderlal Patwa Government Medical College, Mandsaur, based on historical data over a defined period.

Objective: To evaluate the prevalence and trends of the reactivity of screening markers for TTIs in blood donors over a one-year period (December 2023 to November 2024) and to identify any significant patterns associated with demographic variables and donor type.

Methods: A retrospective analysis was conducted of records of 11,924 blood donations. All blood donor samples were screened for TTI markers (HIV, HBV, HCV, and syphilis) using the Roche Cobas e411 immunoassay analyzer (Roche Diagnostics, Rotkreuz, Switzerland) using the electrochemiluminescence immunoassay (ECLIA) technology. Syphilis screening was performed using both treponemal and non-treponemal tests, with results tracked for reactive samples. Donor type (replacement vs. voluntary) and demographic information were analyzed using descriptive statistics and the chi-square test to compare seroreactivity rates.

Results: The overall prevalence of reactive TTI screening markers was 2.40% (287 reactive units). Screening for malaria by the antigenic card test was negative in all 11,924 donor samples. The seroreactivity profile was dominated by syphilis (1.06%), followed by HBV (0.72%), HIV (0.36%), and HCV (0.26%). A statistically highly significant difference was observed between donor groups: the seroreactivity rate was significantly higher in replacement donors (3.20%) compared to voluntary donors (1.87%) (chi-square = 21.61, p < 0.001).

Conclusion: This study provides a detailed assessment of TTI screening reactivity among blood donors in Mandsaur, India. The high seroprevalence of TTI screening markers, particularly in replacement donors, underscores the need for stricter pre-donation screening and increased reliance on voluntary donations to enhance blood safety. Regular monitoring of these markers is essential.

## Introduction

Blood transfusion is a life-saving medical procedure essential for managing conditions ranging from acute blood loss to chronic diseases like thalassemia and cancer. If not monitored, blood transfusions can lead to the transmission of infectious agents, potentially resulting in patient mortality (fatality), chronic morbidity, and significant economic stress [1]. If blood transfusions are not properly monitored, they can spread harmful infections, potentially resulting in patients' mortality (fatality), chronic morbidity, and significant economic stress [1]. The fatality associated with transfusion-transmissible infections (TTIs), particularly HIV and hepatitis B/C, underscores the critical need for robust screening measures, as these infections can lead to life-threatening conditions such as AIDS, liver failure, and hepatocellular carcinoma.

A TTI is any infection transmitted through the administration of blood or blood products. Routinely screened markers include those for hepatitis B virus (HBV), hepatitis C virus (HCV), HIV, and syphilis [2]. These infections can be passed on to recipients even when they do not show symptoms [3,4]. In the context of blood safety in India, other infections are relevant; while Malaria is endemic in certain regions and often screened, organisms like dengue, chikungunya, and *Leishmania* are also endemic in India but not routinely included in mandatory TTI screening panels at all centers. There are higher blood safety challenges compared to places like the US or Europe because of the higher prevalence of certain infections in the general population [5]. The rate of HIV in adults in India was 0.2-0.3% [6]. The average rate of hepatitis B surface antigens in India was 4.7% (ranging from 1% to 3%) [7]. About 4-19.2% of blood donors had HCV infections. While in India, HCV prevalence has been reported as less than 2% [8]. Preventing TTIs is crucial for safe blood transfusions. Using advanced lab tests and methods to kill pathogens is key to reducing the risk of TTIs [9,10].

Background

The prevalence of infectious agents in the general population of developing countries like India often leads to higher blood safety challenges compared to regions like the US or Europe. This is because higher underlying prevalence means more donors may be in the early, undetectable "window period" of infection, increasing transfusion risk even with screening. Furthermore, resource variability in many blood centers can limit the adoption of advanced techniques like nucleic acid testing (NAT), leading to continued dependence on conventional serological assays. Therefore, systematic screening for TTI markers is vital to ensure the safety of the blood supply in resource-constrained settings.

Objective

This study aims to determine the seroprevalence and trends of the reactivity of screening markers for TTIs in blood donors over a specified period at the Indira Gandhi District Hospital associated with Sunderlal Patwa Government Medical College, Mandsaur, India. Additionally, the study seeks to identify significant demographic variables that may correlate with the positivity of TTI screening markers.

## Materials and methods

A retrospective study was conducted based on blood donation records from the Blood Bank of Indira Gandhi District Hospital associated with Sunderlal Patwa Government Medical College, Mandsaur. The records spanned a one-year period from December 2023 to November 2024.

The study included all 11,924 blood donations collected during the study period. Data were collected from the hospital's donor registry, including demographic information and the donor's history. Blood samples were routinely tested for HIV, hepatitis B, hepatitis C, and syphilis. The results of these tests were recorded and analyzed for this study. Donor type was a critical variable collected and analyzed. Voluntary donors (VDs) donate out of altruism without replacement pressure, whereas replacement donors (RDs) donate blood to replace units used by a specific patient (often a family member). Blood donors adhere strictly to the guidelines set by the National Blood Transfusion Council (NBTC), India. Selecting low-risk donors through strict screening involves a thorough pre-donation interview, questionnaire, and physical examination conducted by a qualified medical officer. This process aims to identify and exclude individuals with risk factors (e.g., recent tattoos, fever, and high-risk sexual behavior) before phlebotomy. This initial screening is the first and most critical safeguard, as testing alone cannot detect infections in the window period.

Inclusion criteria included all blood donations recorded during the study period. Exclusion criteria included all blood donations with incomplete or missing records.

All blood donor samples were screened for TTIs using a combination of methods. Viral markers (HIV, HBV, and HCV) and syphilis were tested using the Roche Cobas e411 immunoassay analyzer (Roche Diagnostics, Rotkreuz, Switzerland) using the electrochemiluminescence immunoassay (ECLIA) technology, and malaria was tested using the antigenic card test method. It is important to note that this study reports the prevalence of reactive screening markers only. It does not include results from confirmatory testing or routine NAT platforms, which are often limited in resource-constrained settings. As the Cobas e411 is a commercially licensed laboratory instrument, the relevant operating agreements and quality control documentation are maintained by the blood bank. No scales or scoring systems requiring "free-to-use" verification or specific third-party citation were utilized in this study.

Statistical analysis

The collected data were transcribed into a spreadsheet program and analyzed using SPSS version 26.0 (IBM Corp., Armonk, NY). The analysis specifically addressed the seroreactivity of screening markers and their association with donor characteristics. The methods used are described below.

Seroreactivity Rate Calculation

Descriptive statistics (frequencies and percentages) were used to calculate the seroreactivity rate for each TTI marker (number of reactive samples/total donations) and to describe the demographic characteristics.

Comparative Analysis

The chi-square test was employed to determine if there were statistically significant differences in the TTI screening marker seroreactivity rates between categorical variables, particularly donor type (replacement vs. voluntary).

Significance

A p-value of less than 0.05 (p < 0.05) was considered the threshold for statistical significance in all comparative tests.

## Results

In the present study, records for a total of 11,924 whole blood donations were reviewed. The overall prevalence of TTI screening marker reactivity was 2.40% (287 reactive units). Screening for malaria by antigenic card test was negative in all 11,924 donor samples (0.00% reactivity).

The seroprevalence of screening reactive samples among blood donors is shown in Table 1. The seroreactivity profile was dominated by syphilis (1.06%), followed by HBV (0.72%), HIV (0.36%), and HCV (0.26%).

**Table 1:** Prevalence of reactive TTI screening markers among blood donors. TTI: transfusion-transmissible infection; HBV: hepatitis B virus; HCV: hepatitis C virus.

TTI marker	Dec 2023-Nov 2024 (n = 11,924)	Total (n = 11,924)
HIV	43 (0.36%)	43
HBV	86 (0.72%)	86
HCV	31 (0.26%)	31
Syphilis	127 (1.06%)	127
Malaria	0	0
Total	287 (2.40%)	287

The age distribution of TTI screening participants is shown in Table 2. The highest numbers of HIV and HCV reactive markers were seen among the age group of 18 to 30 years, while syphilis and HBV reactive markers were predominant in the age group of 31 to 40 years. The statistical analysis comparing overall TTI seroreactivity across the four age groups showed no statistically significant difference (chi-square = 5.79, df = 3, p = 0.122).

**Table 2:** Age-wise distribution of TTI screening reactivity among blood donors. Prevalence of TTI according to the given age group. TTI: transfusion-transmissible infection; HBV: hepatitis B virus; HCV: hepatitis C virus.

	18-30	31-40	41-50	>51	Total (n = 11,924)
HIV	21	18	02	02	43
HBV	34	37	14	01	86
HCV	16	08	07	00	31
Syphilis	33	52	36	06	127
Malaria	0	0	0	0	0
TOTAL	104	115	59	9	287

Table 3 shows the seroreactivity of TTI screening markers among blood donors according to their type of donation. The overall TTI screening marker seroreactivity rate was significantly higher in replacement donors (3.20%) compared to voluntary donors (1.87%). This difference was found to be statistically highly significant (chi-square = 21.61, df = 1, p < 0.001).

**Table 3:** Distribution of the seroreactivity of TTI screening markers among blood donors according to their type of donation. TTI: transfusion-transmissible infection; HBV: hepatitis B virus; HCV: hepatitis C virus.

TTI marker	Replacement (n = 4770)	Voluntary (n = 7154)	Total (n = 11,924)
HIV	23 (0.48%)	20 (0.28%)	43
HBV	47 (0.98%)	39 (0.54%)	86
HCV	16 (0.31%)	15 (0.22%)	31
Syphilis	67 (1.40%)	60 (0.83%)	127
Malaria	0	0	0
Total	153 (3.20%)	134 (1.87%)	287 (2.40%)

## Discussion

This study, which reviewed 11,924 donor records, gives a detailed look at TTI screening reactivity among blood donors. The overall TTI seroreactivity rate in our study was 2.40%, which is close to previous findings by Chauhan et al. [11] in India at 2.12%, Mandal et al. [12] at 2.93%, Karmakar et al. [13] at 2.79%, and Koshy et al. [14] at 2.9%. It is crucial to emphasize that these results represent the seroprevalence of screening-reactive samples among donors, not the confirmed prevalence of infection, as some results may be false positives and infections in the window period remain undetected.

Prevalence of TTI seroreactivity

Syphilis was the most frequently reactive marker (1.06%), followed by HBV (0.72%), HIV (0.36%), and HCV (0.26%). The high seroreactivity rate for syphilis may be attributed, at least partially, to the lifelong persistence of treponemal antibodies detected by treponemal screening tests used in the panel, which can indicate past, successfully treated, or current infections. A noteworthy finding is the zero seroreactivity observed for malaria across all 11,924 donor samples, suggesting effective regional control or low donor risk for this vector-borne TTI.

In our study, the HIV seroprevalence was 0.36%. Suresh et al. [15] found similar HIV rates among donors at 0.36%. Sidhu et al. [16] found a lower rate of 0.08%, and Chauhan et al. [11] reported an average of 0.52%. HIV seroprevalence was higher among replacement donors at 0.48%, while it was 0.28% in voluntary donors. Sharma et al. [17] reported 0.45% and 0.32% in replacement and voluntary donors, respectively. Chauhan et al. [11] reported 2.72% and 0.18% in replacement and voluntary donors. HIV can be transmitted during the "window period" even when each unit is tested for HIV antibodies. This can be minimized by selecting low-risk donors through strict screening.

Our study found HBV seroprevalence at 0.72%. Gupta et al. [18] reported a similar rate of 0.66%, and Chauhan et al. [11] reported an average of 0.68%. In our study, HBV prevalence was 0.98% among replacement donors and 0.54% among voluntary donors. Kaur et al. [19] reported 1.07% and 0.65% in replacement and voluntary donors, while Pahuja et al. [20] and Chauhan et al. [11] reported higher rates. The higher HBV seroreactivity observed among replacement donors may be because they often hide health information to get blood for their patients, which risks blood safety.

Our study found HCV seroprevalence at 0.26%. Gupta et al. [18] from Punjab and Chauhan et al. [11] in India reported 0.11%. In our study, HCV seroprevalence was 0.31% among replacement donors and 0.22% among voluntary donors. Kaur et al. [19] reported 0.5% and 0.3%, Sharma et al. [17] reported 0.52% and 0.23%, and Chauhan et al. [11] reported 0.68% and 0.03% in replacement and voluntary donors, respectively.

We found a higher seroprevalence rate of syphilis (1.06%) compared to other TTI screening markers. Pallavi et al. [1] reported a syphilis prevalence of 0.23%. Singogo et al. showed syphilis as the second most common TTI with a prevalence of 3.4% [21]. Another study by Chauhan et al. [11] reported syphilis prevalence of 0.56% in 2018, 0.84% in 2019, and 1.84% in January-February 2020, with an average of 0.81%, which is slightly lower than our current study, except for January-February 2020.

Age distribution

The age distribution of TTI screening markers in our study also aligns with patterns seen in other studies. In our study, HIV- and HCV-positive donors were most commonly found in the 18-30 years age group, while syphilis and HBV-positive donors were predominantly in the 31-40 years age group. Similar trends were observed by Chauhan et al. [11] in India, who reported a higher prevalence of HIV and HCV in younger age groups, which they attributed to higher risk behaviors such as unprotected sex and injection drug use, commonly prevalent among younger adults.

The higher prevalence of syphilis and HBV in the 31-40 years age group in our study is consistent with studies in other settings, such as a study by Chauhan et al. [11] in India, which showed that adults in their 30-40s were more likely to test positive for syphilis and HBV. This may reflect a combination of biological susceptibility, longer exposure to risk factors, and delayed diagnosis and treatment in this age group.

Donor types and TTI seroprevalence

One of the most significant findings in our study was the observation that replacement donors had a significantly higher prevalence of TTI seroreactivity (syphilis, HIV, HCV, and HBV) compared to voluntary donors. This trend (chi-square = 21.61, p < 0.001) has been documented in several studies. For example, in a study by Chauhan et al. [11], replacement donors had a higher prevalence of all TTI screening markers compared to voluntary donors. Replacement donors may have a higher likelihood of being at risk for infections due to the nature of the donor selection process, which sometimes includes individuals with a history of risky behaviors or individuals donating out of necessity rather than altruism. Furthermore, voluntary donors often undergo more stringent pre-donation screening, including questionnaires regarding risk factors, which could lead to a self-selection bias where those at higher risk may refrain from donating voluntarily.

Limitations

This was a retrospective study based solely on donor screening results.

Terminology

The findings represent seroreactivity to TTI screening markers rather than confirmed infections.

Testing Limitations

The study does not include confirmatory testing results or routine NAT data, which is essential to rule out false positives and detect infections in the window period. The higher prevalence of certain infections in the general population in India, coupled with resource limitations, affects the ability to routinely incorporate NAT in many centers, increasing the challenges in blood safety compared to regions with unlimited resources.

Donor Data

The study lacks further characterization of replacement donors, such as their risk profiles and specific motivations, which would enhance the interpretation of the observed risk disparity.

## Conclusions

In conclusion, this study's findings contribute to a clearer understanding of the distribution and seroreactivity patterns of TTIs among blood donors in this geographical area. The relatively high seroprevalence of syphilis, particularly in the 31-40 years age group, and the higher seroprevalence among replacement donors, underscores the need for targeted interventions. Strategies to reduce TTIs in blood donors should focus on improving education and prevention efforts, especially in high-risk age groups and among replacement donors, and centers should strive to incorporate advanced testing, such as NAT, as resources permit, to reduce the risk of window-period transmissions. Further studies with larger sample sizes and a more diverse population are needed to confirm these findings and explore potential contributing factors in greater detail.
